# Clinical presentations, electrophysiologic features, and long-term follow-up in Lambert–Eaton myasthenic syndrome: a series of six patients

**DOI:** 10.3389/fneur.2024.1525155

**Published:** 2024-12-13

**Authors:** Reem M. Alhammad, Yafa Alshamlan, Ruwa Alneseyan, Talal M. Al-Harbi, Ali Alhijab, Mohammed H. Alanazy

**Affiliations:** ^1^Department of Internal Medicine, College of Medicine, King Saud University, Riyadh, Saudi Arabia; ^2^Department of Neurology, King Fahad Specialist Hospital, Dammam, Saudi Arabia

**Keywords:** Lambert–Eaton myasthenic syndrome, neuromuscular junction, voltage-gated calcium channels, small cell lung carcinoma, compound muscle action potential (CMAP)

## Abstract

**Background:**

Lambert–Eaton myasthenic syndrome (LEMS) is an autoimmune disorder of the presynaptic neuromuscular junction associated with antibody mediated dysfunction of voltage-gated calcium channels (VGCCs). LEMS can exist as a paraneoplastic syndrome, paraneoplastic-LEMS (P-LEMS), when associated with tumors, most commonly, small cell lung carcinoma (SCLC) or as a non-paraneoplastic condition (NP-LEMS) when no malignancies are detected.

**Methods:**

A retrospective chart review was conducted in 3 tertiary hospitals in Saudi Arabia for patients diagnosed with LEMS between January 2010 and January 2020. Patients meeting all the following criteria were included: (1) weakness or fatigability of one or more extremity or oculo-bulbar muscles, (2) 60% or higher increment of compound muscle action potential (CMAP) amplitudes immediately following isometric exercise, and (3) positive serum P/Q type VGCC antibodies. Clinical, laboratory, and electrophysiologic features, as well as radiologic imaging modalities performed for tumor screening were reviewed.

**Results:**

The study included six patients diagnosed with LEMS, split evenly between P-LEMS and NP-LEMS. Fatigability, particularly in the lower extremities, and dyspnea on exertion were commonly reported symptoms. Low CMAP amplitudes were more frequently seen in NP-LEMS as compared to P-LEMS when recorded from both abductor pollicis brevis and abductor digiti minimi muscles. An incremental response above 60% in post activation CMAPs was detected at similar rates following variable durations of isometric exercise (10, 15, and 20 s). Tumor types detected in 3 patients with P-LEMS are SCLC, breast carcinoma, colon adenocarcinoma, and prostate acinar adenocarcinoma. Triple malignancy was detected in one patient.

**Conclusion:**

This is the first study to describe clinical and electrophysiologic features of LEMS in an Arab ethnic cohort. Early recognition of LEMS has a significant impact on prognosis, especially given the aggressive nature of associated cancers such as SCLC.

## Introduction

Lambert–Eaton myasthenic syndrome (LEMS) is an autoimmune disorder of the presynaptic neuromuscular junction associated with antibody mediated dysfunction of the alpha 1A subunit of the voltage-gated calcium channel (VGCC). Frequent clinical features include proximal muscle weakness, fatigability, autonomic dysfunction, and reduced or absent deep tendon reflexes (1–5). Neurophysiologic findings include the classic triad of low baseline compound muscle action potential (CMAP) amplitudes, a decremental response on low frequency repetitive nerve stimulation (RNS), and an incremental response following isometric exercise or high frequency RNS (6). LEMS can exist as a paraneoplastic syndrome, i.e., paraneoplastic LEMS (P-LEMS) when an associated malignancy (50–60% of patients) is detected, most commonly, small cell lung carcinoma (SCLC) (7–9), or it may occur without detected malignancies, i.e., non-paraneoplastic LEMS (NP-LEMS) (10). Improving knowledge on the clinical and electrophysiologic features of LEMS can guide early diagnosis and detection of associated malignancies (11). Herein, we aim to assess clinical and electrophysiologic features, as well as tumor types detected in six patients with LEMS. To our knowledge, there are no studies describing LEMS in Arab populations, elucidating these features is highly relevant for improved recognition of this rare disease with profound impacts on patient prognosis and quality of life.

## Methods

### Patients and study design

A retrospective chart review was conducted in three tertiary hospitals in Saudi Arabia to search for cases diagnosed with LEMS between January 2010 and January 2020. Search terms used include Lambert–Eaton myasthenic syndrome, myasthenic syndrome, or presynaptic neuromuscular disorder. Patients were included in analysis if they met all the following criteria: (1) presented with weakness or fatigability of one or more muscle groups in the extremities, ocular, or bulbar muscles, (2) nerve conduction studies reveal 60% or higher increment of CMAP amplitudes immediately following isometric exercise, and (3) positive serum VGCC-P/Q antibodies.

Demographic characteristics, clinical and electrophysiologic features, as well radiologic images performed for the purpose of malignancy screening were reviewed. Laboratory results collected include HbA1c, ESR, CRP, VGCC antibodies, and Sry like high-mobility group box protein 1 antibodies (SOX1) antibodies.

A positive response to immune-modulating or symptomatic therapy was defined as muscle strength improvement by one or more Medical Research Council (MRC) grades in one or muscle groups. Patients with pathologically confirmed malignancy are designated as P-LEMS, while patients with no detected malignancy are designated NP-LEMS.

### Nerve conduction studies

Supramaximal stimulation of the peroneal, tibial, median, and ulnar nerves at distal stimulation sites was performed to record baseline CMAPs from the extensor digitorum brevis (EDB), abductor hallucis longus (AHL), abductor pollicis brevis (APB), and abductor digiti minimi (ADM) muscles, respectively. A single post-activation CMAP was recorded from the ADM and APB muscles following variable durations of isometric exercise (either 10, 15, or 20 s). Low frequency (3 Hz) RNS (LF RNS) of the ulnar and median nerves was performed at distal stimulation sites to record a single train of 5 CMAPs from the ADM and APB muscles, respectively. The baseline-to-negative peak amplitudes of CMAPs before and following isometric exercise were measured. Percent increment in the post-activation CMAP was measured as: 100 X (amplitude highest CMAP-baseline CMAP amplitude/baseline CMAP amplitude). A decremental response to LF RNS was calculated as: 100 X (amplitude baseline CMAP – lowest CMAP amplitude/baseline CMAP amplitude). A positive decremental response was defined as more than 10%. Skin temperature of the extremities was controlled at or above 32°C.

### Statistical analysis

Descriptive statistics are used to summarize baseline characteristics, median time to diagnosis, median time to tumor detection in all LEMS cases, and electrophysiologic findings.

## Results

### Demographic features and clinical findings

An initial chart review captured eight patients from 3 tertiary hospitals with positive P/Q type VGCC antibodies. Two patients were excluded as they showed less than 60% increment in post activation CMAPs. The remaining six patients from 2 tertiary hospitals were included for analysis. Half the patients are male with a median age of 50 years. The most frequently reported symptom is fatigability in the lower extremity, followed by fatigability in upper extremity and dyspnea on exertion. Dry mouth was reported by one patient only. All patients reported dyspnea during exertion or at rest. Forced vital capacity testing was performed in 3 patients, 2 of whom (one NP-LEMS and one P-LEMS) had values less than 80% of predicted for age, gender, height, and weight. Maximal pressures were tested in 2 patients (both NP-LEMS), these show low inspiratory pressures in both (23 and 57% of predicted) with normal maximal expiratory pressures. Other clinical signs and laboratory findings are summarized in Table 1 and Supplementary Table S1.

**Table 1:** Clinical and laboratory features of LEMS patients.

Characteristics	NP-LEMS (*n* = 3)	P-LEMS (*n* = 3)	Total (*n* = 6)
Age at diagnosis, *n*			
<40 years	1	0	1
>40 years	2	3	5
Time from presentation to positive VGCC testing, median (range) months	5 (4.5–8)	15 (7.75–36)	31.5 (4.5–36)
Smoking ever, *n*	1	2	3
Appendicular weakness patterns, *n*			
Proximal muscles of the upper and lower extremities	2	1	3
Proximal and distal muscles of the upper and lower extremities	1	0	1
Proximal and distal lower extremities	0	1	1
Normal strength	0	1	1
DTRs lower limbs, *n*			
Reduced	3	2	5
Absent	0	1	1
DTRs upper limbs, *n*			
Reduced	2	2	4
Absent	1	1	2
Facilitation strength, *n*	1	0	1
Facilitation DTR, *n*	3	2	5
Orthostatic hypotension, *n*	1	0	1
Laboratory tests			
Antibody positivity, *n*/total tested			
AchR	0/2	0	0/2
MUSK	0/2	0	0/2
P/Q VGCC	3/3	3/3	6/6
SOX1	1/1	0	1/1
Laboratory values abnormal, *n*/total tested			
HbA1c (≥5.7%)	1/3	2/2	3/5
ESR > 30 mm/h	1/3	1/2	2/5
CRP > 5 mg/L	1/2	2/2	3/4

Ach, acetylcholine; CRP, C-reactive protein; DTR, deep tendon reflex; ESR, erythrocyte sedimentation rate; HbA1C, glycated hemoglobin; LEMS, Lambert–Eaton myasthenic Syndrome; MUSK, muscle specific kinase; NP-LEMS, non-paraneoplastic LEMS; SOX 1, Sry-like high-mobility group box; P-LEMS, paraneoplastic LEMS; VGCC, voltage gated calcium channel.

### Electrodiagnostic findings

These are summarized in Table 2 and Supplementary Table S2. Low baseline CMAP amplitudes were seen in the majority of upper or lower extremity muscles tested. A decremental response above 10% in CMAP amplitudes following LF RNS was detected in 4 out of 4 tests in the ADM muscle and 2 out of 4 tests in the APB muscle. The ADM muscle was more frequently tested for post activation incremental response in CMAPs as compared to the APB muscle (5 tests vs. 3 tests respectively). An incremental response above 60% in post-activation CMAP amplitudes following isometric exercise was present in all cases and was detected following 10, 15, and 20 s of isometric exercise in two patients each. A positive incremental response (above 60%) in post-activation CMAP amplitudes was detected in 5 out 5 tests in the ADM muscle and 3 out of 3 tests in the APB muscle. An incremental response above 100% in post-activation CMAPs following isometric exercise was present in all but 2 patients. High frequency RNS (HF RNS) (50 Hz) was not performed for any of our patients.

**Table 2:** Nerve conduction study findings in LEMS patients.

Nerve conduction parameter/test	NP-LEMS (*n* = 3)	P-LEMS (*n* = 3)	Total (*n* = 6)
Baseline CMAP amplitudes			
ADM (normal > 6 mV)			
Number with low amplitude/total tested	3/3	2/3	5/6
Median (range), mV	3.5 (2–4)	2 (0.37–6)	2.75 (0.37–6)
APB (normal > 4 mV)			
Number with low amplitude/total tested	2/2	1/2	3/4
Median (range), mV	2.4 (1.7–3.1)	7.08 (1.17–13)	2.4 (1.17–13)
EDB (normal > 2 mV)			
Number with low amplitude/total tested	2/3	1/1	3/4
Median (range), mV	1.5 (0.4–2.5)	1 (1)	1.25 (0.4–2.5)
AHL (normal > 4 mV)			
Number with low amplitude/total tested	3/3	0/1	3/4
Median (range), mV	0.7 (0.42–3)	5 (5)	1.85 (0.42–5)
Muscles tested with 3 Hz repetitive stimulation, *n*			
ADM only	0	2	2
APB only	1	1	2
Both ADM and APB	2	0	2
>10 percent decremental response in CMAP amplitudes following 3 Hz RNS, *n*			
ADM	2	2	4
APB	1	1	2
Muscles tested for post activation increment in CMAPs, *n*			
ADM	3	2	5
APB	2	1	3
>60% incremental response in post- activation CMAP amplitudes following isometric exercise, *n*			
10 s	2	0	2
15 s	0	2	2
20 s	1	1	2
Distribution of post-activation CMAP incremental response in upper extremity muscles (>60%), *n*			
ADM	3	2	5
APB	2	1	3

Ach, acetylcholine; ADM, abductor digiti minimi; APB, abductor pollicis brevis; AHL, abductor hallucis; CMAP, compound muscle action potential; EDB, extensor digitorum brevis; LEMS, Lambert–Eaton myasthenic syndrome; NP-LEMS, non-paraneoplastic LEMS; RNS, repetitive nerve stimulation; P-LEMS, paraneoplastic LEMS.

### Imaging and associated malignancy

**Figure 1:**
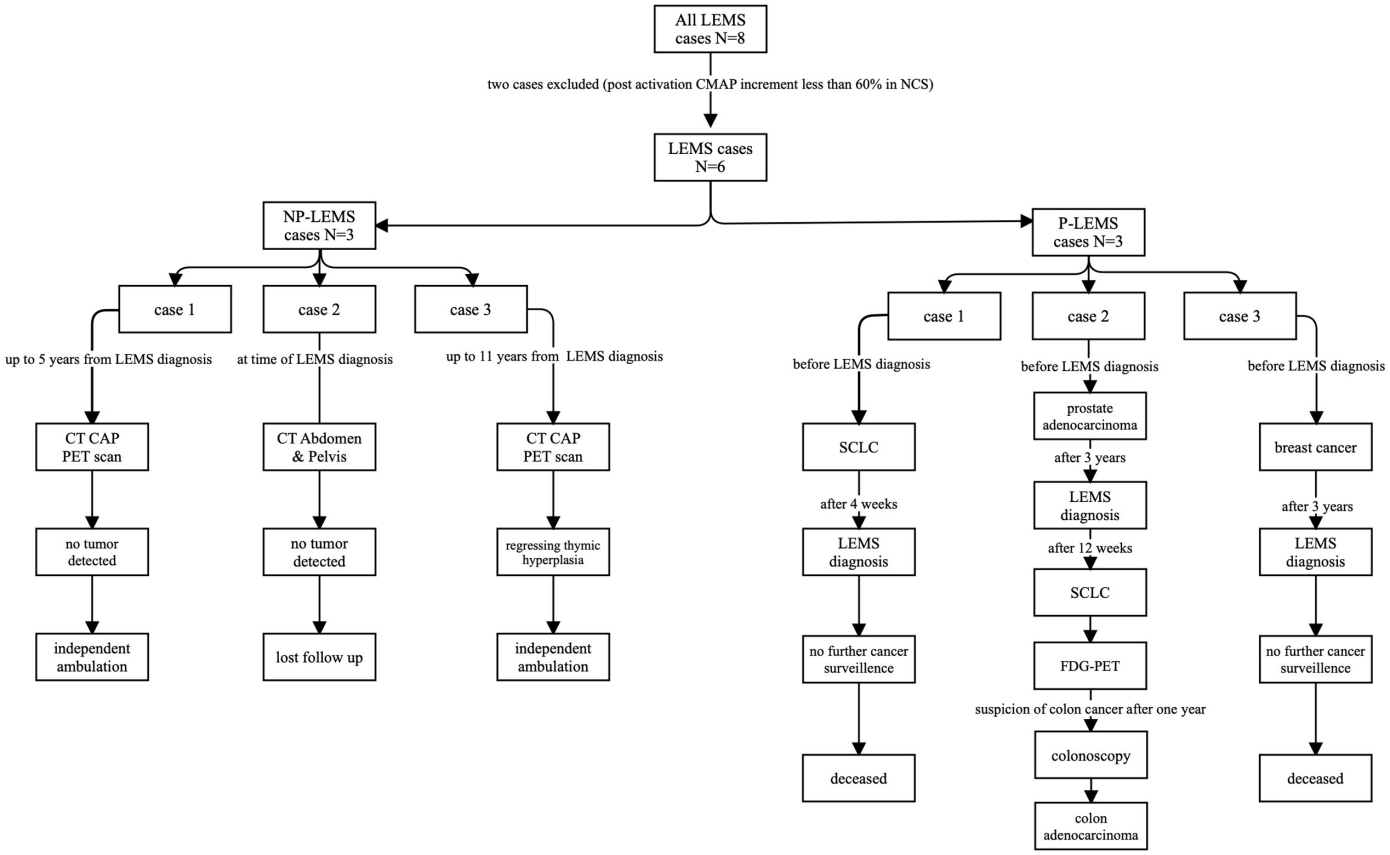
Flowchart of radiologic studies performed, and malignancies detected in our LEMS cohort. LEMS, CT, computerized tomography; CAP, chest-abdomen-pelvis; Lambert–Eaton myasthenic syndrome; NP-LEMS, non-paraneoplastic LEMS; P-LEMS, paraneoplastic LEMS; PET, positron-emission tomography; SCLC, small cell lung carcinoma.

Out of 3 patients with P-LEMS, 2 had been diagnosed with malignancy prior to diagnosis with LEMS (breast cancer, SCLC), time intervals between tumor diagnosis and diagnosis of LEMS were 3 years and 4 weeks, respectively. The third patient with P-LEMS was diagnosed with three separate tumors: prostate acinar adenocarcinoma (gleason score 7 [4 + 3], grade group 3) was diagnosed historically, 3 years prior to the diagnosis with LEMS, then SCLC was detected prospectively by CT scan and fluorodeoxyglucose positron-emission tomography (FDG-PET) scan 12 weeks following diagnosis of LEMS, and the third malignancy was colon adenocarcinoma detected prospectively by FDG-PET 1 year following diagnosis of LEMS. Figure 1 depicts imaging studies performed for tumor screening and malignancies detected in our LEMS cohort.

### Response to symptomatic and immune-modulating therapies

Manual muscle strength testing improvement of >1 MRC grade was documented for one patient following oral prednisolone treatment and for 2 patients following Intravenous Immunoglobulin treatment (Table 3).

**Table 3:** Symptomatic and immune-modulating therapies administered to LEMS patients.

Treatment^§^	NP-LEMS *N* = 3	P-LEMS *N* = 3	MRC improvement of >1 grade with immunosuppressive therapy
3,4, DAP	1	0	0
Pyridostigmine	2	0	0
MMF	1	0	0
Azathioprine	2	0	0
Cyclosporine	1	0	0
Prednisolone (oral)	1	0	1
IVIG	1	1	2

§ More than one therapy given in some patients at variable times.

3,4, DAP, 3,4, diaminopyridine; IVIG, intravenous immunoglobulin; MMF, mycophenolate mofetil; MRC, muscle research council grading scale.

### Long-term follow-up and anti-tumor treatments

Long-term follow up was documented for two patients with NP-LEMS: one patient with associated Hashimoto’s thyroiditis was followed for 5 years, while the other patient with associated SOX1 antibodies was followed for 11 years following LEMS symptom onset. Frequency of various imaging modalities performed for tumor screening, radiologic findings at last follow up visit, and functional outcomes in these two patients are summarized in Supplementary Table S3.

Chemotherapy agents used for the treatment of SCLC were documented for 2 of our P-LEMS patients. These include carboplatin/etoposide for one patient, and Atezolizumab, an anti-programed cell death-ligand 1 (PD-L1) antibody (immune check point inhibitor), in another patient. The latter patient developed symptoms of LEMS prior to initiation of Atezolizumab.

## Discussion

This is the first study to describe the clinical spectrum, neurophysiologic findings, as well as oncologic outcomes in LEMS from an Arab population. To our knowledge, this is also the first study to describe the association of triple malignancy with LEMS and compare the frequency of low baseline CMAP amplitudes in P-LEMS and NP-LEMS.

Similar to prior reports, median age at onset of symptoms in our series was 50 years (11, 12). The most frequent presenting symptoms in our patients is fatigability in proximal upper and lower extremity muscles in addition to dyspnea on exertion. Bulbar symptoms are more prevalent in P-LEMS in our series, consistent with previous studies (12).

Symptoms of autonomic dysfunction are reported in all NP-LEMS patients and were not documented for P-LEMS patients in our cohort. Approximately 80–96% of LEMS cases have autonomic dysfunction which may be the earliest manifestations of this disorder. Dry mouth is the most common symptom followed by erectile dysfunction in men and constipation, while orthostatic hypotension and altered patterns of perspiration are less frequent (12, 13). Dry mouth was reported by only one NP-LEMS patient in our series.

The most frequently affected muscle groups during manual muscle strength assessment in our patients are proximal lower extremity muscles, predominantly involving hip flexion and knee extension, in keeping with previous reports of proximal lower extremity muscle affection in 87% and 90% of NP-LEMS and P-LEMS, respectively (14–16). Deep tendon reflexes were absent or reduced in all our patients, similar to previous studies (13, 17, 18). Postexercise improvement in DTRs was seen in all but one of our patients, more frequently than observed in previous reports (19–21). A single patient in our series (P-LEMS) had normal strength testing with no facilitation detected in muscle strength or DTRs. Such clinical presentations can be diagnostically challenging and require clinical vigilance and a low threshold for electrophysiologic and serological testing for LEMS.

Time from symptom onset to diagnosis was longer in P-LEMS (median 15 months) as compared to NP-LEMS (5 months). This is in contrast to previous reports showing earlier diagnosis in P-LEMS as compared to NP-LEMS with overall intervals from symptom onset to diagnosis ranging between 6 months and 36 years (15, 22).

All of our patients revealed a classic triad of abnormalities in NCS; low baseline CMAP amplitudes, decremental response on low frequency RNS (LF RNS), and incremental response above 60% following isometric exercise. This pattern was the most common of six patterns of NCS abnormalities seen in 71 percent of LEMS cases reported by Oh (6). We used a cutoff of 60% increment or higher in post activation CMAP amplitudes as an inclusion criterion for LEMS based on the findings of Oh et al. (21), who found this cutoff value to be a reasonable alternative to the 100% increment for the diagnosis of LEMS in view of its high diagnostic sensitivity (97%) and specificity (99%). In the current series, an incremental response above 60% in post activation CMAP amplitudes was present in all studies performed on the ADM and APB muscles (5 and 3 tests respectively) and was detected at equal rates following variable durations of isometric exercise, seen in one third of patients following 10, 15, and 20 s of isometric exercise each. Hatanaka and Oh et al. (19) reported post- activation CMAP amplitude increments three times higher following exercise durations of 10-s as compared to 30-s. Increment above 100% was observed in post-activation CMAP amplitudes in all but 2 of our patients (Supplementary Table S2). Maddison et al. (23) studied the distribution of RNS abnormalities in 10 patients with LEMS in the ADM, APB, anconeus, biceps, and trapezius muscles and concluded that the most sensitive muscles for detecting the characteristic abnormalities (low CMAP amplitudes and increment above 100% after 10 s exercise) were the ADM and anconeus muscles. This contrasts to the higher sensitivity of APB muscle in detecting decremental responses in Myasthenia Gravis (24, 25). The presence of normal baseline CMAP amplitudes and lack of incremental response following isometric exercise or HF RNS in Myasthenia Gravis differentiates this disorder from LEMS (6).

Similar to findings reported by Maddison et al. (23), we detected a decremental response following LF RNS more frequently in the ADM muscle as compared to the APB muscle (4 out of 4 compared to 2 out of 4 tests respectively). To our knowledge, no studies have compared the frequency of low baseline CMAP amplitudes in P-LEMS and NP-LEMS. We observed low baseline CMAP amplitudes more frequently in our NP-LEMS patients as compared to P-LEMS when recorded from both APB (2/2 tested vs. 1/2 tested respectively) and ADM (3/3 tested vs. 2/3 tested respectively) muscles. These observations are limited by the small number of cases in our cohort.

All of our patients are VGCC-P/Q seropositive as this was an inclusion criterion, these antibodies are highly sensitive and specific, detected in 90% of LEMS, more frequently in P-LEMS (26–28). None of our patients were tested for VGCC-N. In a study of neurologic accompaniments of 236 VGCC seropositive patients, LEMS was present in 2.5% of patients, all of whom were seropositive for VGCC-P/Q and none had detectable VGCC-N antibodies (28). The significance of VGCC-P/Q or VGCC-N antibodies must be interpreted in the context of clinical and paraclinical findings as these antibodies were detected in diverse autoimmune neurologic phenotypes other than LEMS and in 4% of neurologically asymptomatic patients with lung cancer (28). A single patient with NP-LEMS in our cohort was tested for SOX1 antibody and was seropositive. SOX1 antibodies are more frequent in P-LEMS but have been detected in up to 6% of NP-LEMS (5).

Half of our patients with LEMS had associated tumors (P-LEMS), with a predominance in males, similar to previous reports (12, 14, 29, 30). Tumors detected in our cohort are similar to those previously reported in association with LEMS (26, 31–33): SCLC (detected in two patients), breast carcinoma (one patient), colon adenocarcinoma (one patient), and prostate acinar adenocarcinoma (one patient). The presence of triple malignancy in association with LEMS in one of our patients is not previously reported to our knowledge and underscores individualized cancer surveillance in P-LEMS. Tumor screening can also be guided by the Dutch-English LEMS Tumor Association Prediction (DELTA-P) score, developed and validated by Titulaer et al. (34). No malignancies were detected following comprehensive cancer surveillance in the single patient in our series with NP-LEMS who was seropositive for SOX1 up to 11 years from the diagnosis of LEMS. This patient had evidence of thymic hyperplasia from the initial PET-CT image which later regressed and was not detectable in the last FDG-PET scan performed (Supplementary Table S3).

Treatment of LEMS involves treating the underlying tumor in P-LEMS as well as symptomatic and immune-modulating therapies for both P-LEMS and NP-LEMS. Symptomatic treatment with 3,4-diaminopyridine (3,4-DAP) in LEMS patients showed clinically significant improvements in QMG scores (physician-rated quantitative assessment score) as well as improved resting CMAP amplitudes compared to placebo in a recent meta-analysis (35). 3,4-DAP is a potassium channel blocker that prolongs depolarization of the presynaptic terminal by increasing the influx of calcium through the VGCC, thus increasing the release of acetylcholine manifested as improved muscle function and autonomic symptoms (1, 36). Acetylcholinesterase inhibitors such as pyridostigmine have been used in combination with 3,4 DAP with some studies showing improved benefit above 3,4 DAP monotherapy (37).

Immune-modulating treatments prescribed to our patients include prednisolone, cyclosporine, azathioprine, and IVIG. IVIG is mostly used to treat exacerbations and is equally effective for seronegative and seropositive LEMS (38). The combination of prednisolone and azathioprine improved muscle strength and resting CMAP amplitudes recorded from the ADM in a combined retrospective and prospective study of 47 patients with NP-LEMS (39).

A single patient in our cohort was treated with Atezolizumab, an immune check point inhibitor (ICI), for the management of SCLC. Symptoms of LEMS in this patient developed prior to initiating Atezolizumab. LEMS has been reported to develop as an immune related adverse event (irAE) secondary to multiple ICIs (nivolumab, ipilimumab, 668 atezolizumab, and pembrolizumab) with some cases showing clinical improvement following treatment with oral prednisolone or IVIg (40–43). The safety of ICI administration to patients with LEMS is unknown with reports of both worsening and stability of neurologic symptoms in patients with pre-existing LEMS treated with ICIs (44, 45).

The LEMS has a considerable impact on health status with up to 75% of patients reporting partial or total restrictions in their activities of daily living (ADLs) (46). The two patients with NP-LEMS in our series had suboptimal MG-ADL scores at last follow-up visits (scores of 3 and 11 at 5 and 11 years following LEMS diagnosis respectively) but remained independent for self-care and ambulation, consistent with overall stable disease course seen in long-term observational studies (47).

Limitations of this study include the retrospective design, the small sample size, and tertiary care setting of participating centers, all limiting generalizability of our findings. The small number of cases could reflect the condition’s true rarity or under-recognition of LEMS symptoms within the limited population screened in this study.

In conclusion, this is the first report of LEMS in patients of Arab ethnicity elucidating detailed clinical and NCS findings. Early clinical recognition of LEMS can guide tumor screening and early tumor detection, profoundly impacting therapy and prognosis. This is especially crucial in SCLC, an aggressive tumor with poor prognosis. Studies with larger numbers of patients are needed to assess efficacy of various symptomatic and immunosuppressive therapies.

## Data Availability

The original contributions presented in the study are included in the article/Supplementary material, further inquiries can be directed to the corresponding author.
