# Supplementary material for: Clinical presentations, electrophysiologic features, and long-term follow-up in Lambert–Eaton myasthenic syndrome: a series of six patients
Source: Front Neurol. 2024 Dec 13;15:1525155. doi: 10.3389/fneur.2024.1525155 (PMC11671300; doi:10.3389/fneur.2024.1525155)
Supplement: Supplementary file 1 [file Table_1.DOCX]

**Supplementary Table 1. Symptoms and signs in LEMS**

| **Symptoms** | **NP-LEMS(n=3)** | **P-LEMS(n=3)** | **Total (n=6)** |
| --- | --- | --- | --- |
| **Double vision** | 1 | 1 | 2 |
| **Dysphagia** | 1 | 2 | 3 |
| **Dyspnea on exertion** | 2 | 3 | 5 |
| **Dyspnea at rest** | 1 | 2 | 3 |
| **Fatigability upper extremities** | 3 | 3 | 5 |
| **Fatigability lower extremities** | 3 | 3 | 6 |
| **Upper extremity weakness only** | 1 | 0 | 1 |
| **Lower extremity weakness only** | 0 | 1 | 1 |
| **Upper and lower extremity weakness** | 3 | 0 | 3 |
| **Dry mouth** | 1 | ND | 1 |
| **Micturition difficulties** | 2 | ND | 2 |
| **Persistent diarrhea** | 2 | ND | 2 |
| **Heat intolerance** | 2 | ND | 2 |
| **Orthostatic intolerance** | 2 | ND | 2 |
| **Early satiety** | 2 | ND | 2 |
| **Signs, n** |  |  |  |
| **Ptosis** | 1 | 0 | 1 |
| **Ophthalmoplegia** | 0 | 0 | 0 |
| **Facial weakness** | 0 | 0 | 0 |
| **Neck flexion weakness** | 0 | 0 | 0 |
| **Neck extension weakness** | 0 | 0 | 0 |

NP-LEMS= non-paraneoplastic LEMS; P-LEMS=paraneoplastic LEMS

**Supplementary Table 2. Distribution of decremental response following 3 Hz repetitive nerve simulation and incremental response following isometric exercise among upper extremity muscles in LEMS**

| **Age** | **LEMS**  **type** | **APB** | | **Duration of Isometric Exercise**  **(Seconds)** | **ADM** | |
| --- | --- | --- | --- | --- | --- | --- |
|  |  | **CMAP Decrement**  **(% of amplitude) ^§^** | **CMAP Increment**  **(% of amplitude)** |  | **CMAP Decrement**  **(% of amplitude) ^§^** | **CMAP Increment**  **(% of amplitude)** |
| 50-60 | NP-LEMS | NT | NT | 10 | 30 | 71 |
| 20-30 | NP-LEMS | 0 | 330 | 10 | 15 | 440 |
| 70-80 | NP-LEMS | 15 | 100 | 20 | 5 | 180 |
| 50-60 | P-LEMS | NT | NT | 15 | 18 | 300 |
| 40-50 | P-LEMS | NT | NT | 20 | 20 | 200 |
| 60-70 | P-LEMS | 20 | 70 | 15 | NT | NT |

^§^ Following low frequency (3 HZ) repetitive nerve stimulation

ADM=Abductor digiti minimi; APB= Abductor Pollicis Brevis; CMAP=Compound Muscle Action Potential; NP-LEMS= non-paraneoplastic LEMS; P-LEMS= paraneoplastic LEMS; NT= not tested

**Supplementary Table 3. Frequency of tumor screening modalities, radiologic and clinical features in 2 patients with NP-LEMS at last follow- up.**

|  | **NP-LEMS case 1** | **NP-LEMS case 2** |
| --- | --- | --- |
| **Age at LEMS diagnosis** | 50-60 | 20-30 |
| **Associated autoimmune conditions** | Hashimoto’s thyroiditis | None |
| **Antibodies detected** | P/Q VGCC, TPO, Thyroglobulin | P/Q VGCC, SOX1 |
| **DELTA-P score** | 1 | 0 |
| **Imaging frequency** |  |  |
| **CT chest, abdomen, pelvis** | q 6 months in first year, then once every 2 years | q 6 months in first year, then once every 2 years |
| **PET scan** | q 6 months in first year, then once every 2 years | q 6 months in first year, then once every 2 years |
| **Mammogram** | Annual | Annual |
| **Ultrasound** | Annual | Annual |
| **Time interval from LEMS diagnosis to last imaging study, years** |  |  |
| **CT chest, abdomen, pelvis** | 5 | 11 |
| **PET scan** | 5 | 11 |
| **Radiologic findings in last imaging study** |  |  |
| **CT chest, abdomen, pelvis** | Uterine fibroids | No abnormality |
| **FDG-PET scan** | No FDG avid lesions | Regression of thymic hyperplasia |
| **Mammogram** | Benign nodule (BI-RADS 3) | NT |
| **Breast ultrasound** | NT | Normal |
| **Last follow up visit following LEMS diagnosis, years** | 5 | 11 |
| **Treatment at last follow up** | Pyridostigmine, Azathioprine, IVIg for exacerbations | 3,4 DAP 10mg daily, cyclosporine150 mg daily, prednisolone 5mg |
| **MGQOL-15R** | 11 | 13 |
| **MG-ADL** | 3 | 11 |
| **Ambulation** | Independent | Independent |

CT= Computerized tomography; DELTA-P= Dutch-English LEMS Tumor Association Prediction score; FDG- MG-ADL= Myasthenia Gravis activities of daily living score; MGQOL-15R= Myasthenia Gravis Quality of Life 15 item revised score; NP-LEMS= non-paraneoplastic LEMS; PET= positron Emission tomography; P/Q VGCC= P/Q type voltage gated calcium channel antibody; SOX1= Anti-Sry-like high-mobility group box 1; TPO= Thyroid peroxidase
